# CPP-PNA Conjugate-Mediated Inhibition of *pdxA* Gene Impairs Vitamin B6 Biosynthesis and Growth in *Acinetobacter baumannii*

**DOI:** 10.3390/ijms27020584

**Published:** 2026-01-06

**Authors:** Wook-Jong Jeon, Ju Hui Seo, Yoo Jeong Kim, Song-mee Bae, Dong Chan Moon

**Affiliations:** Division of Antimicrobial Resistance Research, National Institute of Health, Korea Disease Control and Prevention Agency, 187 Osongsaengmyeong 2-ro, Osong-eup, Heungdeok-gu, Cheongju-si 28159, Chungcheongbuk-do, Republic of Korea; finalfate@korea.kr (W.-J.J.); hi0327@korea.kr (J.H.S.); yyjj0203@korea.kr (Y.J.K.); songmee@korea.kr (S.-m.B.)

**Keywords:** cell-penetrating peptides, antisense oligonucleotide, peptide nucleic acid, *Acinetobacter baumannii*

## Abstract

*Acinetobacter baumannii* represents a critical-priority organism due to its multidrug resistance. The emergence of carbapenem-resistant strains poses a major clinical challenge, underscoring the urgent need for novel antibacterial agents with alternative mechanisms. As peptide nucleic acids (PNAs) have recently gained attention as antisense therapeutics, we aimed to validate their potential as novel antimicrobial strategies against multidrug-resistant *A. baumannii*. We synthesized a cell-penetrating peptide (CPP)–PNA conjugate targeting *pdxA*, an essential gene involved in vitamin B6 biosynthesis. Among several candidate genes tested, the *pdxA*-targeting PNA exhibited the strongest inhibitory activity, achieving complete growth suppression of *A. baumannii* at 1.56 μM. Although quantitative real-time polymerase chain reaction did not reveal significant reductions in *pdxA* transcript levels, ELISA quantification revealed an approximately 80% reduction in intracellular vitamin B6, indicating translational inhibition rather than mRNA degradation. The *pdxA*-targeting CPP–PNA showed negligible activity against other Gram-negative or Gram-positive species, indicating high target specificity; no detectable cytotoxicity in human cells was observed even at relatively high concentrations. CPP–PNA conjugates targeting *pdxA* interfere with vitamin B6 biosynthesis, leading to growth inhibition of *A. baumannii*. These findings support PNA as a promising antisense antimicrobial platform that inhibits multidrug-resistant *A. baumannii* by blocking vitamin B6 biosynthesis.

## 1. Introduction

*Acinetobacter baumannii* is an opportunistic, Gram-negative bacterium that has emerged as one of the most problematic pathogens in hospital environments. It is an aerobic, non-fermentative, rod-shaped microorganism commonly detected in soil, water, and medical facilities [1,2]. Due to its remarkable resilience and ability to adhere to abiotic surfaces, *A. baumannii* often causes healthcare-associated infections such as ventilator-associated pneumonia, wound infections, bacteremia, and urinary tract infections [3].

This organism, belonging to the ESKAPE group of pathogens, frequently exhibits multidrug resistance and extensive drug resistance [4]. The growing prevalence of antibiotic resistance in *A. baumannii* poses a serious threat to global public health. In recognition of this, the World Health Organization (WHO) listed carbapenem-resistant *A. baumannii* as a critical-priority pathogen in 2024, underscoring the urgent need for alternative therapeutic options [5].

Most currently available antibiotics act on a limited essential bacterial processes, including cell wall synthesis, protein translation, nucleic acid replication, transcription, and membrane integrity [6]. However, these conventional targets are becoming increasingly ineffective against multidrug-resistant pathogens. Therefore, identifying new targets specific to bacteria is a key step toward developing effective antimicrobial strategies [7].

Traditionally, antimicrobial peptides (AMPs) have been recognized for their rapid bactericidal activity through direct disruption of bacterial membranes or increasing membrane permeability [8]. However, these membrane-targeting mechanisms are often associated with nonspecific toxicity to host cells, limited target specificity, and the potential for resistance development [9]. To overcome these limitations, peptide nucleic acids (PNAs) have emerged as promising antisense agents, offering highly specific inhibition of bacterial genes while minimizing off-target effects, and have consequently attracted considerable research interest [10]. PNAs are synthetic analogs of DNA in which the sugar–phosphate backbone is replaced by a neutral, peptide-like structure. This modification confers enhanced binding affinity, sequence specificity, and resistance to enzymatic degradation compared to natural nucleic acids, and also provides high stability in biological environments [11]. When conjugated with cell-penetrating peptides (CPPs), PNAs can efficiently cross bacterial membranes and block target gene expression through antisense mechanisms [10]. For example, the CPP sequence (KFF)_3_K has been shown to enhance both the cellular uptake and antibacterial efficacy of PNAs directed against essential genes such as *acpP* in *Escherichia coli* [12]. Similarly, a (KFF)_3_K–PNA conjugate targeting the essential gene *carA* in *A. baumannii* demonstrated strong growth-inhibitory effects [13].

Targeting bacterial-specific metabolic pathways represents a promising approach for the development of selective therapeutics with a reduced risk of resistance [14]. In particular, vitamin biosynthesis pathways are of great interest, as they are crucial for bacterial survival yet absent in human cells, making them attractive candidates for novel antimicrobial targets [15]. The de novo biosynthetic pathway for vitamin B6, which culminates in the production of pyridoxal 5′-phosphate (PLP)—an essential cofactor for numerous enzymatic reactions in amino-acid, carbohydrate, and lipid metabolism—is indispensable for bacterial growth and pathogenicity [16,17]. In many pathogenic species, genetic disruption of this pathway results in profound growth impairment and markedly reduced virulence [18,19]. Notably, mammals lack a de novo PLP biosynthesis pathway, relying instead on dietary intake or microbiota-derived vitamin B6. This fundamental difference renders the bacterial PLP pathway a highly selective and promising target for antibacterial drug development [20,21].

In this study, CPP–PNA conjugates were designed and synthesized to target various genes involved in the vitamin B6 biosynthesis pathway of *A. baumannii*. By evaluating whether these PNAs suppress the expression of target genes and inhibit vitamin B6 biosynthesis, we aimed to validate their potential as a novel antisense-based antimicrobial strategy and to establish a foundation for developing new therapeutic alternatives against multidrug-resistant *A. baumannii* infections.

## 2. Results

### 2.1. Inhibition of Bacterial Cell Growth by CPP–PNA Conjugates

The antibacterial activities of the synthesized CPP–PNA conjugates targeting five putative vitamin B6 biosynthesis genes were evaluated against *A. baumannii* ATCC 17978 by quantifying colony-forming units 4 h after treatment with various concentrations of each PNA (Table 1). Among the five CPP–PNA conjugates designed to target different genes, the conjugate targeting *pdxA* showed the strongest growth inhibitory effect, demonstrating significant activity even at a concentration of 25 μM. The CPP–PNA conjugate targeting *A1S_2516* also showed growth inhibition, but only at 50 μM. In contrast, conjugates targeting *A1S_2617, A1S_2637*, and *A1S_3106* did not exhibit any noticeable inhibitory effect even at 50 μM.

Colony-forming units (CFUs) observed following CPP–PNA treatment. A total of 10 μL of *A. baumannii* ATCC 17978 at an optical density at 600 nm (OD_600_) of 0.8 was incubated with various concentrations of PNAs. After 4 h of incubation, the bacterial culture was spread on Mueller–Hinton agar plates. The plates were incubated at 37 °C for 24 h, and the number of colonies was counted. All PNAs used in this study were designed based on the genome sequence of *A. baumannii ATCC 17978* (CP000521), and their exact sequences are listed in Table S1. This test was performed in duplicate, and all instances of ‘-’ in the table denote values that were too numerous to be counted.

### 2.2. Effect of PNA Length on Inhibitory Activity

The binding site and nucleotide length of PNAs are important factors affecting their efficacy. To identify the PNA with the best effect, we designed diverse PNAs and performed a minimum inhibitory concentration (MIC) test (Table 2). The designed PNA sequence is shown in Table S1. The effect of the binding site was tested first, and the effect of length variation was subsequently examined with the most effective site. PNAs designed to target the ribosome-binding site (RBS) and start codon had the lowest MIC (6.25 μM) compared with those designed to target only the start (12.5 μM) or stop codons (25 μM). In length-variation assays, shorter PNA length was associated with a reduction in MIC. The 10-mer PNA demonstrated an MIC of 1.56 μM, representing a fourfold increase in antibacterial efficacy compared with the 13-mer PNA (6.25 μM).

### 2.3. Species-Specific Activity of PNA

Due to their high sequence specificity, CPP–PNA conjugates act only on bacteria that carry the matching target sequence and are able to take up the conjugate into their cells. To confirm species specificity, the most potent PNA, PNA-*pdxA*6, was tested against *Pseudomonas aeruginosa*, *Escherichia coli*, and *Staphylococcus aureus*, all of which lack a perfectly matched target sequence (Tables S2–S4). Against *E. coli* and *P. aeruginosa*, the antimicrobial activity was much weaker than that observed in *A. baumannii*, and notably, no activity was detected against the Gram-positive bacterium *S. aureus* (Table 3).

### 2.4. Evaluation of CPP–PNA Conjugate Cytotoxicity

**Figure 1:**
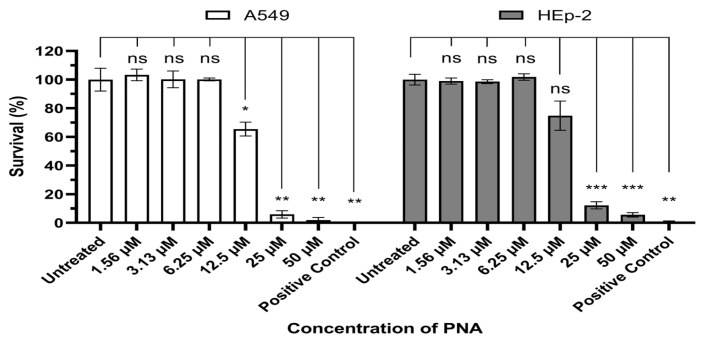
Evaluation of PNA-pdxA6 cytotoxicity in A549 and HEp-2 cell. Each cell was incubated for 24 h under various concentrations of CPP–PNA, after which cell viability was quantified using the Cell Counting Kit-8 assay. Data is expressed as percentages relative to the untreated control, and each concentration was tested in triplicate. A 10% DMSO solution was used as the positive control. Statistical comparisons between the negative control and each treatment group were performed using Brown-Forsythe and Welch ANOVA tests. ns: not significant, *: *p* < 0.05, **: *p* < 0.01, ***: *p* < 0.001. PNA, peptide nucleic acids.

In this study, we used (KFF)_3_K, an amphipathic peptide composed of repeated lysine (K) and phenylalanine (F) residues. Cytotoxicity was evaluated in vitro using A549 and HEp-2 cells (Figure 1). Significant cytotoxicity was observed at concentrations above 12.5 μM (IC_50_/MIC = 8.01) and 25 μM (IC_50_/MIC = 16.03) in A549 cells and Hep-2 cells, whereas no reduction in cell viability was detected at lower concentrations. These results were consistent across both A549 and HEp-2 cells.

### 2.5. Antisense Effect of CPP-PNA Conjugates for pdxA Inhibits Vitamin B6 Synthesis

**Figure 2:**
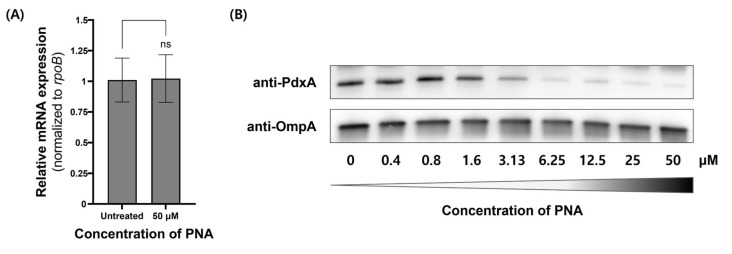
*pdxA* gene expression in *A. baumannii* ATCC 17978 after PNA-*pdxA* treatment. *A. baumannii* ATCC 17978 at approximately 1.0 × 10^9^ CFU mL^−1^ was cultured for 4 h with 0 μM or 50 μM of PNA-pdxA6. (**A**) The mRNA transcript levels of the *pdxA* genes. (**B**) The relative expression levels of the *pdxA* genes were analyzed using quantitative real-time PCR. The *rpoB* gene was used as a control. The original Western blot images are provided in Figure S7 of the Supplementary Materials. ns: not significant. PNA, peptide nucleic acids.

**Figure 3:**
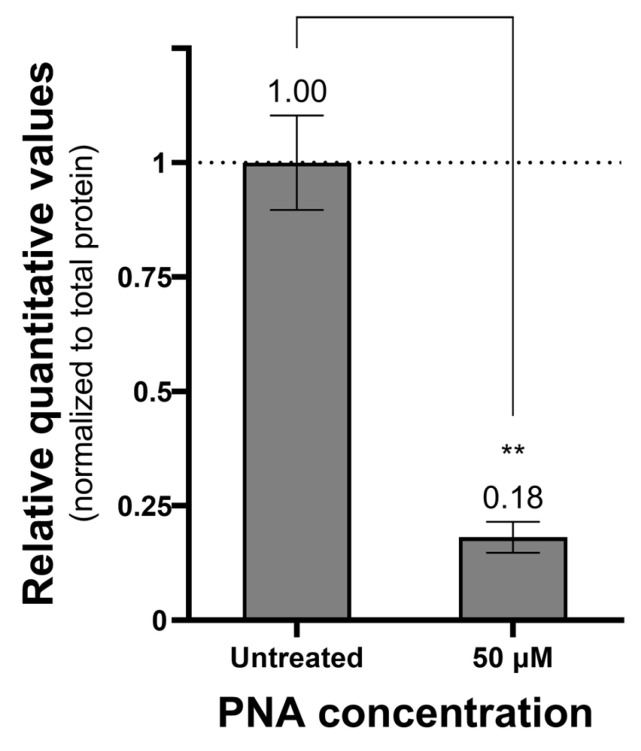
Amount of vitamin B6 in PNA-treated cells. Relative quantitative value of vitamin B6 in cells treated with PNA compared to untreated cells. Cells treated with 0 or 50 μM PNA concentration for 4 h were centrifuged, and only the pellet was collected and ultrasonicated. The prepared samples were quantified for vitamins B6 and total proteins in the cells using a vitamin B6 ELISA kit and a Bradford assay kit. The measured vitamin B6 amounts were normalized to the total protein amount. **: *p* < 0.01. PNA, peptide nucleic acids.

The antisense activity of CPP–PNA conjugates targeting the *pdxA* gene was evaluated using real-time polymerase chain reaction (PCR) and Western blot to measure mRNA and protein levels, respectively (Figure 2). At a concentration of 50 μM, PNA-*pdxA6* caused no significant change in mRNA levels. In contrast, protein levels decreased in a CPP–PNA concentration-dependent manner, with a maximum reduction of approximately 95%. To further confirm gene silencing, intracellular vitamin B6 levels were measured. Measurement using a vitamin B6 enzyme-linked immunosorbent assay (ELISA) kit showed that treated cells had more than an 80% reduction in intracellular vitamin B6 compared with untreated controls (Figure 3).

## 3. Discussion

Similarly to PNAs, phosphorodiamidate morpholino oligomers (PMOs) are synthetic antisense oligonucleotides that bind complementarily to DNA or RNA to suppress gene expression [22]. In eukaryotic systems, their low toxicity and high stability have enabled clinical applications, with FDA approval reported for therapies such as those for Duchenne muscular dystrophy (DMD) [23,24]. However, direct comparative studies targeting the same genes have shown that CPP–PNA conjugates generally exhibit lower minimum inhibitory concentrations (MICs) and stronger antimicrobial activity than CPP–PMO conjugates [25].

To date, various cell-penetrating peptides (CPPs), including Tat, penetratin, transportan, and arginine-rich peptides, have been reported for intracellular delivery of nucleic acid–based therapeutics [26]. Among these candidates, the (KFF)_3_K sequence was selected as the CPP for conjugation with PNA, as it is the most widely used in antisense studies against bacteria and has consistently demonstrated reproducible antimicrobial activity. (KFF)_3_K is a positively charged amphipathic peptide that has been shown in multiple studies to effectively traverse both the outer and inner membranes of Gram-negative bacteria, thereby delivering PNAs into the cytoplasm. In addition, its short peptide length facilitates synthesis, reduces variability in delivery efficiency, and minimizes cytotoxicity [25,27]

In this study, a (KFF)_3_K–PNA conjugate was designed and optimized to target essential genes involved in the bacterial vitamin B6 (PLP) biosynthesis pathway. Among the five initial candidate genes selected for PNA design, three (*A1S_2617*, *A1S_2637*, and *A1S_3106*) showed no antimicrobial activity. Of the two genes that did exhibit antimicrobial effects (*A1S_2516* and *pdxA*), *pdxA* demonstrated the strongest inhibitory effect and was therefore chosen as the final target gene for PNA development.

To optimize the *pdxA*-targeting PNA, 13-mer PNAs were designed to bind various regions of the gene, including the RBS, start codon, and stop codon. MIC analysis revealed that targeting both the RBS and start codon region (−9 to +4) yielded the most potent effect, with an MIC of 6.25 μM. This finding is consistent with previous reports indicating that PNAs blocking the RBS and start codon effectively inhibit translation initiation [28,29]. As an additional optimization strategy, the length of the PNA sequence was systematically adjusted, and corresponding MIC values were evaluated. Reducing the length from 13-mer to 12-mer resulted in a twofold decrease in MIC, while no change was observed at 11-mer. However, a 10-mer PNA showed a fourfold decrease in MIC compared to the 13-mer, indicating that the 10-mer achieves an optimal balance between target specificity and intracellular uptake efficiency in *A. baumannii*. This result is consistent with previous findings [27,29].

To assess species specificity, MIC assays were performed using the *pdxA*-targeting PNA conjugate against *S. aureus* ATCC 29213, *E. coli* ATCC 25922, and *P. aeruginosa* ATCC 27853. Although all four species possess the *pdxA* gene, sequence variations suggest that the designed PNA would be specific to *A. baumannii*, which was confirmed by the MIC results. In the Gram-positive bacterium *S. aureus*, no growth inhibition was observed even at concentrations above 50 μM. This outcome is likely due to both the sequence specificity of the PNA and the reduced efficacy of the cell-penetrating peptide (KFF)_3_K in Gram-positive bacteria compared to Gram-negative ones [29]. In contrast, the Gram-negative species, *E. coli* and *P. aeruginosa,* showed modest growth inhibition at 12.5 μM, yet this was more than eightfold higher than the MIC observed in *A. baumannii* (1.56 μM), indicating a marked difference in susceptibility. The slight inhibitory effects in *E. coli* and *P. aeruginosa* are presumed to result from nonspecific interactions between the (KFF)_3_K–PNA conjugate and partially homologous gene sequences, consistent with previous studies involving Gram-negative bacteria [13].

Cytotoxicity analysis revealed that the concentration at which toxicity was observed in mammalian cells was more than eightfold higher than the concentration required for antimicrobial activity, indicating a relatively favorable safety profile. The observed toxicity appears to be attributed to membrane interactions of the CPP rather than the PNA itself. Positively charged CPPs such as (KFF)_3_K, Tat, and penetrating have been reported to disrupt eukaryotic cell membranes at high concentrations [30,31]. Therefore, future improvements should focus on modifying the charge distribution or structural features of CPPs to reduce cytotoxicity while maintaining efficient bacterial delivery.

Gene expression analysis showed an approximately 95% reduction in protein levels, whereas quantitative real-time PCR revealed no significant changes at the mRNA level. This suggests that the PNA used in this study effectively suppresses gene expression primarily at the translational stage rather than at the transcriptional stage. This finding is consistent with previously reported results for a PNA targeting *GyrA* in *E. coli* [32]. PNAs generally act either by inducing mRNA degradation through RNaseH activation or by sterically blocking mRNA translation and processing. In bacteria, antisense targeting has predominantly been performed using PNAs and PMO oligomers, which do not activate RNaseH and are therefore known to exert their effects mainly through translational inhibition [33,34].

Vitamin B6 is an essential metabolite that functions as a cofactor in numerous enzymatic reactions, including those involved in amino acid, lipid, and carbohydrate metabolism [35]. Therefore, inhibition of its biosynthetic pathway can simultaneously affect multiple physiological metabolic processes in bacteria. ELISA analysis of vitamin B6 levels revealed a significant decrease following *pdxA* suppression, confirming that the observed antimicrobial effect results from direct blockade of the vitamin B6 biosynthesis pathway rather than nonspecific stress or off-target effects.

Overall, this study demonstrates that targeting *pdxA* with PNA effectively disrupts vitamin B6 biosynthesis and inhibits bacterial growth. These findings highlight the potential of PNAs as a selective antimicrobial strategy capable of precisely silencing essential bacterial genes. Future research should prioritize optimizing cell-penetrating peptides to improve intracellular delivery and stability in serum and against enzymatic degradation, validating their efficacy in in vivo infection models, and exploring combination therapies with conventional antibiotics to enhance antimicrobial activity.

## 4. Materials and Methods

### 4.1. Bacterial Strains and Culture Conditions

*A. baumannii* ATCC 17978, *S. aureus* ATCC 29213, and *P. aeruginosa* ATCC 27853 were cultivated in Tryptic soy agar (#500505, Synergy Innovation, Seongnam-si, Republic of Korea) or Tryptic soy broth (#211825, BD, Franklin Lakes, NJ, USA) at 37 °C. For MIC testing, cells were incubated overnight in Mueller–Hinton agar (#500108, Synergy Innovation) at 37 °C.

### 4.2. Design and Synthesis of CPP-PNA Conjugates

All PNAs used in this study were designed based on the genome of *A. baumannii* ATCC 17978 (GenBank accession, CP033110.1), with detailed sequences provided in Table S1. Each PNA was commercially synthesized and purified by PANAGENE, Inc. (Daejeon, Republic of Korea), and subsequently conjugated to the (KFF)_3_K cell-penetrating peptide. The purity and molecular weight of the conjugates were determined and confirmed using high-performance liquid chromatography and matrix-assisted laser desorption/ionization time-of-flight mass spectrometry (Figures S1–S6).

### 4.3. Determination of Minimal Effective Concentrations of CPP-PNA Conjugates

Cultures of *A. baumannii* ATCC 17978 were grown to an OD_600_ of 0.8, after which 10 µL of the culture (1 × 10^7^ CFU/mL) was inoculated into each well of a 96-well plate. CPP–PNA conjugates dissolved in Mueller–Hinton broth were added to achieve final concentrations of 0, 1.56, 3.13, 6.25, 12.5, 25, and 50 µM per well, with the total volume adjusted to 100 µL. The 96-well plate was incubated at 37 °C for 4 h, after which 100 µL of the bacterial suspension from each treated well was spread onto two Mueller–Hinton agar plates (50 µL per plate). The inoculated plates were then incubated at 37 °C for 24 h to allow bacterial growth and colony formation.

### 4.4. Determination of the MICs of CPP-PNA Conjugates

*A. baumannii* ATCC 17978 was cultured overnight and then diluted in Mueller–Hinton broth to yield approximately 5.0 × 10^4^ CFU/mL per well in a 96-well plate. CPP–PNAs were added to achieve final concentrations of 0, 1.56, 3.13, 6.25, 12.5, 25, and 50 µM, with the total sample volume adjusted to 100 µL per well. The prepared 96-well plate was incubated at 37 °C for 16–20 h, and the concentrations at which bacterial growth was inhibited were determined. The broth microdilution method used in this assay was performed according to Clinical and Laboratory Standards Institute guidelines [36].

### 4.5. Quantitative Real-Time PCR

*A. baumannii* ATCC 17978, at approximately 1.0 × 10^9^ CFU/mL, was cultured in Mueller–Hinton broth at 37 °C with 50 μM of CPP-PNA conjugates. After 4 h of incubation, the total bacterial RNAs were extracted using the RNeasy mini kit (#74104, QIAGEN, Hilden, Germany). The resultant RNAs were treated with RNase-free DNase Set (#79254, QIAGEN) to eliminate all contaminating DNAs. The total RNAs were reverse-transcribed into cDNA using RNA-to-cDNA EcoDry^TM^ Premix (#639546, Takara, San Jose, CA, USA), and the quantitative real-time PCR was performed using 7500 Fast Real-Time PCR System (Applied Biosystems, Foster City, CA, USA) and THUNDERBIRD^TM^ Next SYBR^®^ qPCR Mix (#TOQPX-201, TOYOBO, Osaka, Japan).

The thermal conditions are as follows: a preincubation at 95 °C for 20 s, followed by 40 cycles with denaturing at 95 °C for 3 s and elongation at 60 °C for 30 s. The *rpoA* gene was used as a control. All the primers in this study are listed in Table 4. The gene expression levels were analyzed using the comparative delta-delta Ct (2^−ΔΔCt^) method [37].

### 4.6. Western Blot

*A. baumannii* ATCC 17978, at approximately 1.28 × 10^8^ CFU/mL, was cultured in Mueller–Hinton broth (#275730, BD, Le Pont de Claix, France) at 37 °C with 50 μM of CPP-PNA conjugates. After 4 h of incubation, bacterial cells were collected by centrifugation. Cell pellets were resuspended in 1× Laemmli sample solution (#1610737, Bio-Rad, Hercules, CA, USA) and heated at 95 °C for 5 min. Then, proteins were separated by 12% Mini-PROTEAN TGX Precast Protein Gels (#4561045, Bio-Rad) and transferred onto a PVDF membrane using Trans-Blot Turbo Transfer System (#1704150 and #1704156, Bio-Rad).

To reduce non-specific binding, the membrane was blocked by incubation in EveryBlot Blocking Buffer (#12010020, Bio-Rad) for 5 min. The blocked membrane was then incubated for 1 h at 25 °C, with the appropriate primary antibodies, polyclonal anti-PdxA antibody (1:1000) or Acinetobacter OmpA Monoclonal Antibody (MA5-47562, 1:2000, Invitrogen, Waltham, MA, USA). The anti-PdxA antibody was custom-produced using recombinant PdxA protein purified by Engitein (Seoul, Republic of Korea), and subsequently generated as a polyclonal antibody in rabbits through YNTOAB (Seongnam-si, Republic of Korea). The membrane was washed four times with phosphate-buffered saline (PBS-T) and then probed with appropriate secondary antibodies, Immun-Star Goat Anti-Mouse-HRP Conjugate (#1705047, 1:2000, Bio-Rad) or Immun-Star Goat Anti-Rabbit-HRP Conjugate (#1705046, 1:2000, Bio-Rad), and incubated for 1 h at room temperature. After washing the membrane with 1× PBS-T five times, the membrane was overlaid with ECL-SuperKine™ West Femto Maximum Sensitivity Substrate (BMU102-EN, Abbkine, Atlanta, GA, USA) for anti-FLAG immunoblotting or Miracle-Star™ Western blot detection system (#16028, iNtRON, Seongnam-si, Republic of Korea) for anti-RpoA immunoblotting, and chemiluminescence was imaged using the ChemiDoc Go Imaging System (Bio-Rad).

### 4.7. Measurement of Vitamin B6 Levels Using ELISA

*A. baumannii* ATCC 17978 at approximately 1.0 × 10^8^ CFU/mL was cultured in Mueller–Hinton broth at 37 °C. A 1.6 mL aliquot of culture was transferred to an E-tube, centrifuged, and the pellet resuspended in 1.6 mL of PBS or PBS adjusted to 50 μM PNA. Samples were incubated at 37 °C for 4 h. After PNA treatment, the samples were centrifuged at 6000× *g* for 3 min, the supernatant was discarded, and the pellet was resuspended in 1 mL of PBS. Subsequently, samples were lysed using an ultrasonicator (VCX750, Sonics&Materials, Newtown, CT, USA) and debris was removed by centrifugation (16,000× *g* for 30 min at 4 °C). For the whole cell lysate, Vitamin B6 ELISA was performed three times using the Vitamin B6 ELISA Kit (abx150386, Abbexa, Cambridge, UK). Samples were normalized using the Bradford Protein Assay (T9310A, Takara).

### 4.8. Cell Toxicity Assay

Cell toxicity determination was performed using A549 and HEp-2 cells. A549 and Hep-2 cells were grown in Roswell Park Memorial Institute 1640 medium (#CM058-050, GenDEPOT, Altair, TX, USA) and Dulbecco’s Modified Eagle Medium (#12430-054, Gibco, Grand Island, NY, USA) supplemented with 10% fetal bovine serum and 1% Penicillin-Streptomycin (#15140-122, Gibco), respectively.

A549 cells (5 × 10^3^ cells/cm^2^) and Hep-2 cells (1 × 10^4^ cells/cm^2^) were seeded in the wells of a 96-well plate and incubated at 37 °C with 5% CO_2_. After 24 h, the cells were washed with 1× PBS and exposed to 50, 25, 12.5, 6.25, 3.13, 1.56, and 0 μM of CPP-PNA conjugates for 24 h at 37 °C with 5% CO_2_. Additionally, 10% DMSO was used as a positive control. Absorbance at 450 nm was used as a measure of cell viability, and the absorbance 450 nm measurement was performed according to the Cell Counting Kit-8 assay kit (#96992, Sigma, St. Louis, MO, USA). Fluorescence signals were measured on an Infinite M200 microplate reader (TECAN, Männedorf, Switzerland). Each test was performed in biological triplicate, and data were presented as mean ± standard error.

### 4.9. Statistical Analysis

Statistical analyses were performed using GraphPad Prism version 8.4.3. Each experiment included at least three independent biological replicates. Data are presented as mean  ±  standard deviation. For in vitro experiments, statistical significance was assessed using one-way ANOVA. Significance levels were defined as: * *p*  <  0.05, ** *p*  <  0.01, *** *p*  <  0.001, and **** *p*  <  0.0001.

## 5. Conclusions

This study demonstrates that *pdxA* is a highly effective antisense target for disrupting the vitamin B6 biosynthesis pathway in *A. baumannii*. By optimizing both the target region and PNA length, the (KFF)_3_K–PNA conjugate developed herein achieved strong antimicrobial activity, species specificity, and low cytotoxicity at therapeutically relevant concentrations. Furthermore, this PNA exerted potent inhibitory effects at the translational level without inducing mRNA degradation, and suppression of *pdxA* led to a significant decrease in intracellular vitamin B6 levels. These findings confirm that the antimicrobial effect arises from direct blockade of the vitamin B6 biosynthesis pathway rather than nonspecific binding or stress responses.

Taken together, this study highlights the high potential of PNA-based gene silencing technology as a selective antimicrobial strategy against *A. baumannii*. Nevertheless, cell-penetrating carriers remain a critical challenge in this field. Further optimization of CPPs or the development of novel carriers will be essential to reduce cytotoxicity while enhancing specificity and uptake efficiency for clinical application of PNAs. In addition, validation of efficacy in in vivo infection models and exploration of combination strategies with existing antibiotics will be necessary to further expand the clinical applicability of antisense antimicrobials.

## Figures and Tables

**Table 1:** MBC results of CPP–PNA conjugates targeting the vitamin B6 metabolism pathway in *A. baumannii*.

Target Gene	Function	CPP-PNA Sequence	CPP–PNA Treatment Concentrations (μM)				
			50	25	12.5	6.25	0
*A1S_2516*	Pyridoxine 5′-phosphate synthase	(KFF)_3_K-CCATAAAAAAATC	0 CFUs	1100 CFUs	-	-	-
*A1S_2617*	phosphoserine aminotransferase	(KFF)_3_K-GCATGATTTTTCC	-	-	-	-	-
*A1S_2637*	erythronate-4-phosphate dehydrogenase	(KFF)_3_K-TCATGGTCTGCAA	-	-	-	-	-
*pdxA*	4-hydroxythreonine-4-phosphate dehydrogenase	(KFF)_3_K-TCACCCCCGACAC	0 CFUs	0 CFUs	256 CFUs	-	-
*A1S_3106*	1-deoxy-D-xylulose-5-phosphate synthase	(KFF)_3_K-ACATATGTGGCAA	-	-	-	-	-

MBC, minimum bactericidal concentration; PNA, peptide nucleic acids; CPP, cell-penetrating peptides.

**Table 2:** MIC of diverse CPP–PNAs targeting the *pdxA* gene in *A. baumannii*.

Gene (Target Region)	Location	CPP	PNA Sequence	PNA Length (mer)	MIC (μM)
*pdxA* (Start codon)	+1 to +13	(KFF)_3_K	GCAGCACAATCAC	13	12.5
*pdxA* (Stop codon)	+963 to +975	(KFF)_3_K	TTAACTCGCTGCC	13	25.0
*pdxA* (RBS + Start codon)	−9 to +4	(KFF)_3_K	TCACCCCCGACAC	13	6.25
*pdxA* (RBS + Start codon)	−9 to +3	(KFF)_3_K	CACCCCCGACAC	12	3.13
*pdxA* (RBS + Start codon)	−9 to +2	(KFF)_3_K	ACCCCCGACAC	11	3.13
*pdxA* (RBS + Start codon)	−9 to +1	(KFF)_3_K	CCCCCGACAC	10	1.56

*A. baumannii* inhibition effects of *pdxA*-targeted PNA. *A. baumannii* ATCC 17978 was incubated with various concentrations of CPP-PNA conjugates, and the minimum inhibitory concentration (MIC) was measured. This test was performed in duplicate. PNA, peptide nucleic acids; CPP, cell-penetrating peptides.

**Table 3:** MIC of CPP–PNAs targeting the *pdxA* gene in diverse bacterial species.

PNA Name (Target Region)	CPP	PNA Sequence	MIC (μM)		
			*E. coli* ATCC 25922	*S. aureus* ATCC 29213	*P. aeruginosa* ATCC 27853
PNA-*pdxA*6 (RBS + Start codon)	(KFF)_3_K	CCCCCGACAC	12.5	>50	12.5

*A. baumannii* inhibition effects of *pdxA*-targeted PNA in diverse bacterial species. *E. coli* ATCC25922, *S. aureus* ATCC29213, and *P. aeruginosa* ATCC 27853 were incubated with various concentrations of CPP-PNA conjugates and measured minimum inhibitory concentration (MIC). This test was performed in duplicate. PNA, peptide nucleic acids; CPP, cell-penetrating peptides.

**Table 4:** Primer sequences used for Quantitative Real-Time PCR in this study.

Primer Name	Oligonucleotide Sequence (5′ to 3′)	Reference
pdxA-F	GTCGATCATGGTACTGCTCTG	This study
pdxA-R	AACTCGCTGCCAAGGATAAA	This study
rpoB-F	TGACTCTGGTGTGTGTGTAATC	This study
rpoB-R	CACCTGCAACCATTTCATCTTC	This study

## Data Availability

The original contributions presented in this study are included in the article/Supplementary Materials. Further inquiries can be directed to the corresponding author.

## Supplementary Materials

The following supporting information can be downloaded at: https://www.mdpi.com/article/10.3390/ijms27020584/s1.

## Abbreviations

The following abbreviations are used in this manuscript:

CPP	cell-penetrating peptides
ELISA	enzyme-linked immunosorbent assay
MIC	minimum inhibitory concentration
PNA	peptide nucleic acids
PLP	pyridoxal 5′-phosphate
PBS	phosphate-buffered saline
