# Supplementary material for: CPP-PNA Conjugate-Mediated Inhibition of pdxA Gene Impairs Vitamin B6 Biosynthesis and Growth in Acinetobacter baumannii
Source: Int J Mol Sci. 2026 Jan 6;27(2):584. doi: 10.3390/ijms27020584 (PMC12841171; doi:10.3390/ijms27020584)

Table S1. Sequences of the PNAs used and their binding sites within the target genes.

PNA name	Target gene	PNA sequence	Location	Reference
PNA-2516	*A1S_2516*	KFFKFFKFFK-CCATAAAAAAATC	-9 to +4 (RBS+Start codon)	This study
PNA-2617	*A1S_2617*	KFFKFFKFFK-GCATGATTTTTCC	-9 to +4 (RBS+Start codon)	This study
PNA-2637	*A1S_2637*	KFFKFFKFFK-TCATGGTCTGCAA	-9 to +4 (RBS+Start codon)	This study
PNA-3106	*A1S_3106*	KFFKFFKFFK-ACATATGTGGCAA	-9 to +4 (RBS+Start codon)	This study
PNA-*pdxA*	*pdxA*	KFFKFFKFFK-TCACCCCCGACAC	-9 to +4 (RBS+Start codon)	This study
PNA-*pdxA*2	*pdxA*	KFFKFFKFFK-GCAGCACAATCAC	+1 to +13 (Start codon)	This study
PNA-*pdxA*3	*pdxA*	KFFKFFKFFK-TTAACTCGCTGCC	+963 to +975 (Stop codon)	This study
PNA-*pdxA*4	*pdxA*	KFFKFFKFFK-CACCCCCGACAC	-9 to +3 (RBS+Start codon)	This study
PNA-*pdxA*5	*pdxA*	KFFKFFKFFK-ACCCCCGACAC	-9 to +2 (RBS+Start codon)	This study
PNA-*pdxA*6	*pdxA*	KFFKFFKFFK-CCCCCGACAC	-9 to +1 (RBS+Start codon)	This study

All PNAs were designed based on *Acinetobacter baumannii* ATCC 17978 (CP000521). PNA, peptide nucleic acid

Table S2. Potential Gene Binding Sites in the *E. coli* ATCC25922 Genome (CP009072) for PNA-pdxA6.

No.	Gene	Function	Locus tag
1	-	tRNA-Pro	DR76_93
2	-	bacterial regulatory helix-turn-helix, lysR family protein	DR76_4622

Table S3. Potential Gene Binding Sites in the *S. aureus* ATCC29213 Genome (CP123086) for PNA-pdxA6.

No.	Gene	Function	Locus tag
No homologous gene exists.			

Table S4. Potential Gene Binding Sites in the *P. aeruginosa* ATCC27853 Genome (CP015117) for PNA-pdxA6.

No.	Gene	Function	Locus tag
1	-	Non-coding DNA sequences	
2	-	tRNA-Ser	A4W92_07085
3	-	Non-coding DNA sequences	
4	-	formyltetrahydrofolate deformylase	A4W92_15840
5	-	terminase	A4W92_23135
6	-	RND transporter	A4W92_30230
7	-	AraC family transcriptional regulator	A4W92_31010

Figure S1. Certificate of Analysis (COA) summary for *pdxA*- targeting CPP-PNA conjugates.

PNA

Sample No (Name) : **01 (vita-13m-RBS / C25024)**

Lot No : **250975**

N-term C-term

Sequence : KFFKFFKFFK-tca ccc ccg aca c

Amount

OD (260nm) : 2.3 **nmole:** 20.0 **Molecular Weight:** 4806.0 **Weight:** 95.9 **μg**

(Weight was calculated from OD value & Extinction coefficient at 260nm, ε_{260} $^{+}$= 114.4 mL/(μmol x cm))

+ ε_{260}(a) = 13.7mL/(μmolexcm), ε_{260}(g) = 11.7mL/(μmolexcm), ε_{260}(c) = 6.6mL/(μmolexcm), ε_{260}(t) = 8.8mL/(μmolexcm),

Analysis

HPLC

Instrument : Agilent 1100 Series (Agilent Technologies)

Purity : 99.9%

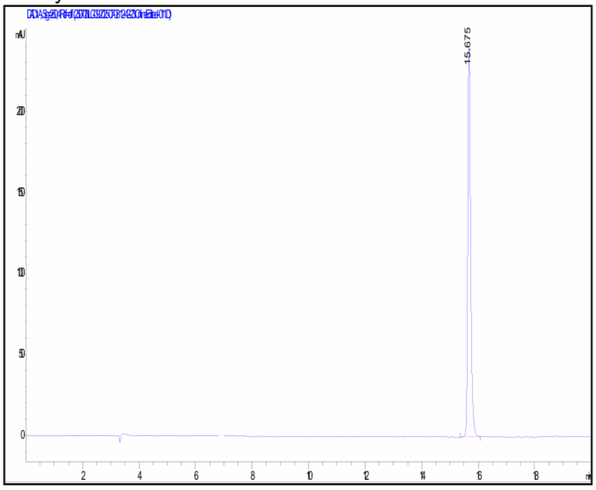

MALDI MS

Instrument: AXIMA-Assurance (Shimadzu Biotech)

MS Found (M+1) : 4807.1

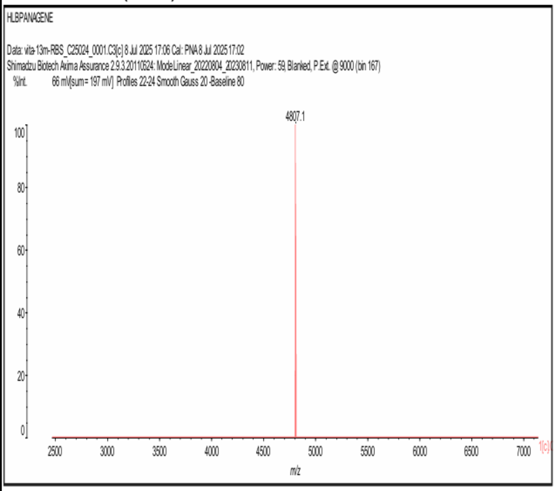

Figure S2. Certificate of Analysis (COA) summary for *pdxA*- targeting CPP-PNA conjugates.

PNA

Sample No (Name) : 02 (vita-13m-Start / C25025)

Lot No : 250976

N-term C-term

Sequence : KFFKFFKFFK-gca gca caa tca c

Amount

OD (260nm) : 2.7 **nmole:** 20.0 **Molecular Weight:** 4894.1 **Weight:** 97.9 µg

(Weight was calculated from OD value & Extinction coefficient at 260nm, $\varepsilon_{260}{}^{+}$= 133.7 mL/(µmol x cm))

+ ε_{260}(a) = 13.7mL/(µmolexcm), ε_{260}(g) = 11.7mL/(µmolexcm), ε_{260}(c) = 6.6mL/(µmolexcm), ε_{260}(t) = 8.8mL/(µmolexcm),

Analysis

HPLC

Instrument : Agilent 1100 Series (Agilent Technologies)

Purity : 99.9%

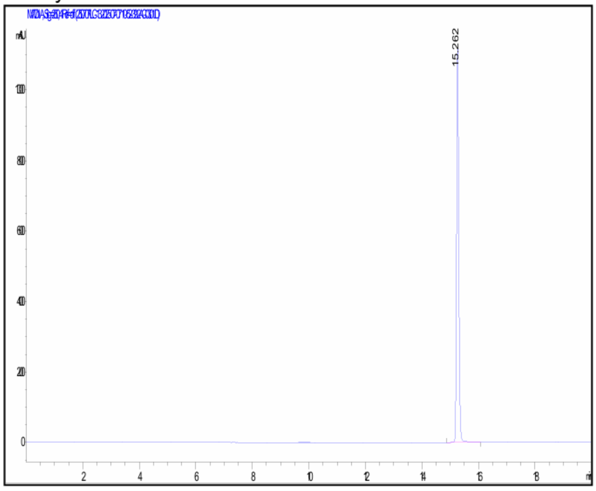

MALDI MS

Instrument: AXIMA-Assurance (Shimadzu Biotech)

MS Found (M+1) : 4894.3

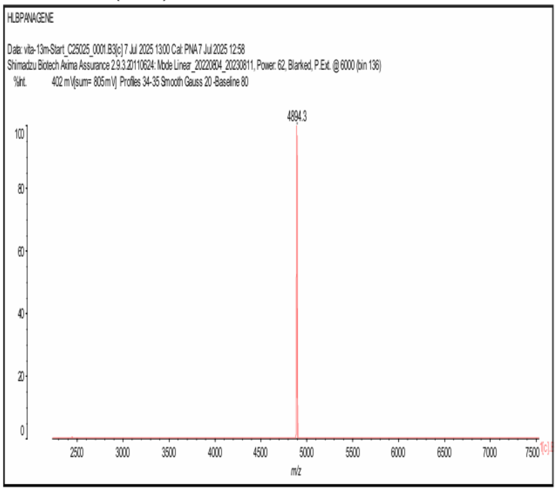

Figure S3. Certificate of Analysis (COA) summary for *pdxA*- targeting CPP-PNA conjugates.

PNA

Sample No(Name): 03 (vita-13m-Stop / C25026)

Lot No : 250977

N-term C-term

Sequence : KFFKFFKFFK-tta act cgc tgc c

Amount

OD(260nm): 2.4 **nmole:** 20.0 **Molecular Weight:** 4867.1 **Weight:** 97.2 µg

(Weight was calculated from OD value & Extinction coefficient at 260nm, $\varepsilon_{260}{}^{+}$= 119.0 mL/(µmol x cm))

+ ε_{260}(a) = 13.7mL/(µmolexcm), ε_{260}(g) = 11.7mL/(µmolexcm), ε_{260}(c) = 6.6mL/(µmolexcm), ε_{260}(t) = 8.8mL/(µmolexcm),

Analysis

HPLC

Instrument : Agilent 1100 Series (Agilent Technologies)

Purity : 99.3%

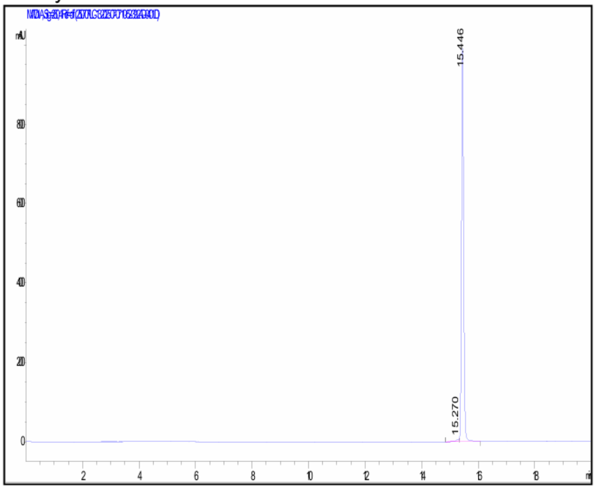

MALDI MS

Instrument: AXIMA-Assurance (Shimadzu Biotech)

MS Found (M+1) : 4867.9

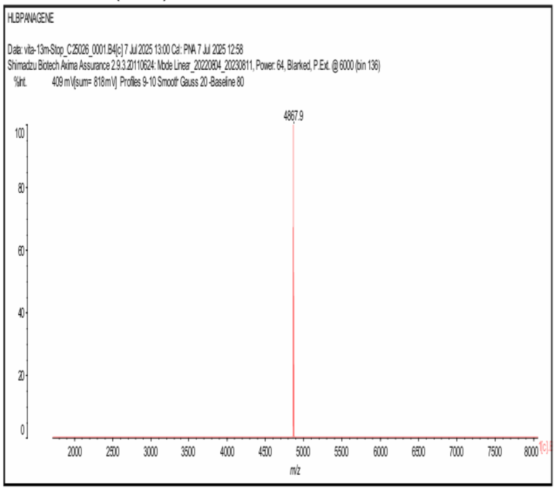

Figure S4. Certificate of Analysis (COA) summary for *pdxA*- targeting CPP-PNA conjugates.

PNA

Sample No(Name): 04 (vita-12m / C25027)

Lot No : 250978

Sequence : N-term KFFKFFKFFK-cac ccc cga cac C-term

Amount

OD(260nm): 2.1 **nmole:** 20.0 **Molecular Weight:** 4539.8 **Weight:** 91.0 μg

(Weight was calculated from OD value & Extinction coefficient at 260nm, ε_{260} $^{+}$= 105.6 mL/(μmol x cm))

+ ε_{260}(a) = 13.7mL/(μmolexcm), ε_{260}(g) = 11.7mL/(μmolexcm), ε_{260}(c) = 6.6mL/(μmolexcm), ε_{260}(t) = 8.8mL/(μmolexcm),

Analysis

HPLC

Instrument : Agilent 1100 Series (Agilent Technologies)

Purity : 99.9%

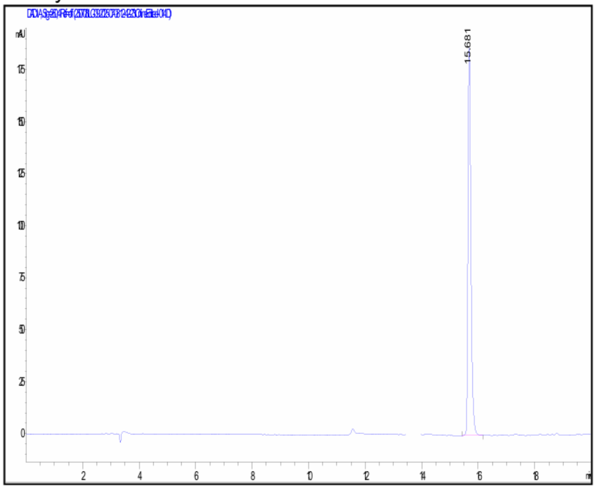

MALDI MS

Instrument: AXIMA-Assurance (Shimadzu Biotech)

MS Found (M+1) : 4541.0

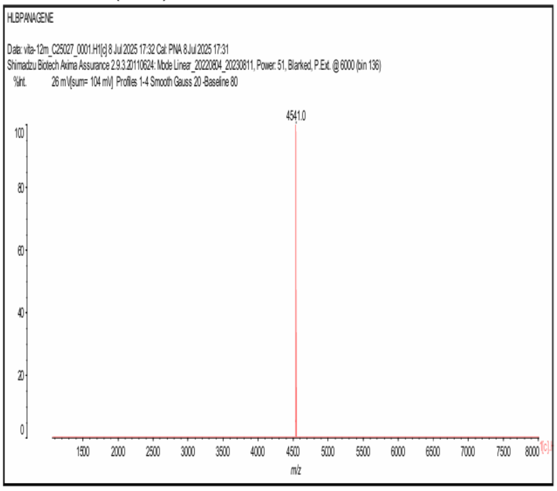

Figure S5. Certificate of Analysis (COA) summary for *pdxA*- targeting CPP-PNA conjugates.

PNA

Sample No (Name) : 05 (vita-11m / C25028)

Lot No : 250979

N-term C-term

Sequence : KFFKFFKFFK-acc ccc gac ac

Amount

OD (260nm) : 2.0 **nmole:** 20.0 **Molecular Weight:** 4288.5 **Weight:** 85.9 µg

(Weight was calculated from OD value & Extinction coefficient at 260nm, ε_{260} $^{+}$= 99.0 mL/(µmol x cm))

+ ε_{260}(a) = 13.7mL/(µmolexcm), ε_{260}(g) = 11.7mL/(µmolexcm), ε_{260}(c) = 6.6mL/(µmolexcm), ε_{260}(t) = 8.8mL/(µmolexcm),

Analysis

HPLC

Instrument : Agilent 1100 Series (Agilent Technologies)

Purity : 99.9%

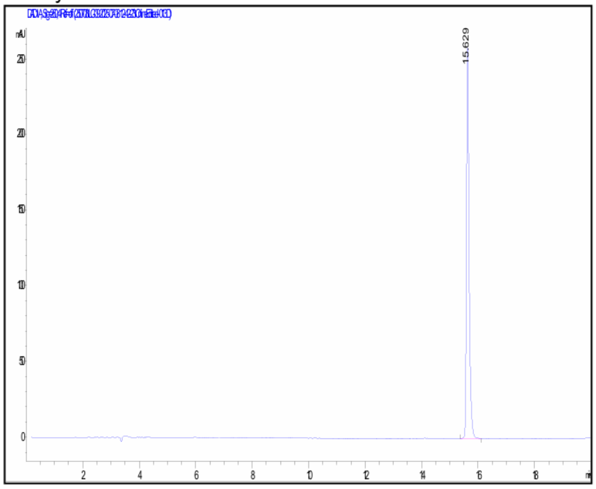

MALDI MS

Instrument: AXIMA-Assurance (Shimadzu Biotech)

MS Found (M+1) : 4288.7

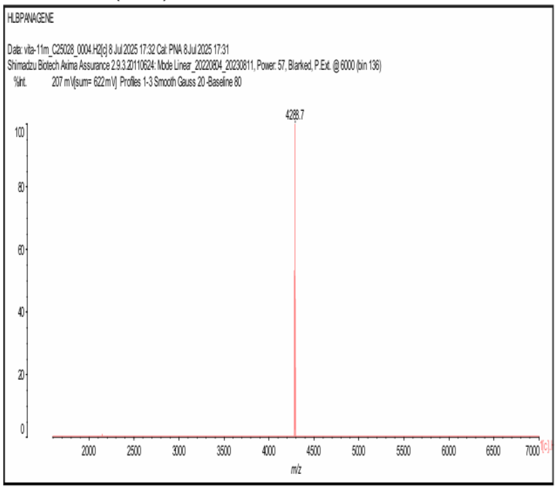

Figure S6. Certificate of Analysis (COA) summary for *pdxA*- targeting CPP-PNA conjugates.

PNA

Sample No(Name): 06 (vita-10m / C25029)

Lot No : 250980

N-term C-term

Sequence : KFFKFFKFFK-ccc ccg aca c

Amount

OD(260nm): 1.7 **nmole:** 20.0 **Molecular Weight:** 4013.3 **Weight:** 80.1 μg

(Weight was calculated from OD value & Extinction coefficient at 260nm, ε_{260} $^{+}$= 85.3 mL/(μmol x cm))

+ ε_{260}(a) = 13.7mL/(μmolexcm), ε_{260}(g) = 11.7mL/(μmolexcm), ε_{260}(c) = 6.6mL/(μmolexcm), ε_{260}(t) = 8.8mL/(μmolexcm),

Analysis

HPLC

Instrument : Agilent 1100 Series (Agilent Technologies)

Purity : 99.9%

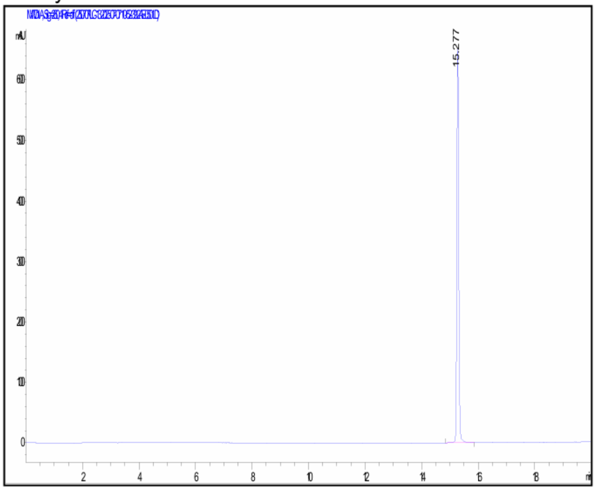

MALDI MS

Instrument: AXIMA-Assurance (Shimadzu Biotech)

MS Found (M+1) : 4013.8

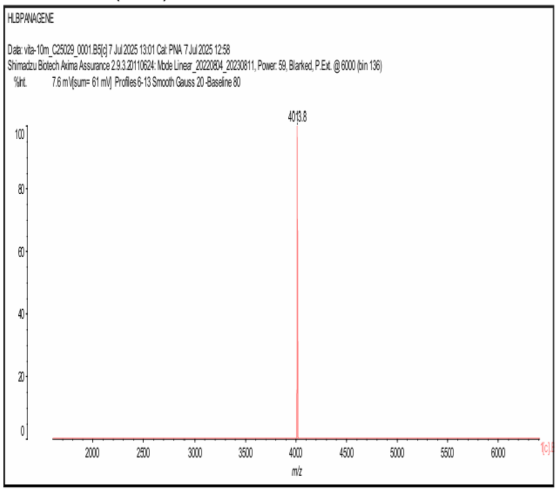

Figure S7. *pdxA*-targeting CPP–PNA conjugate–treated western blot original image.

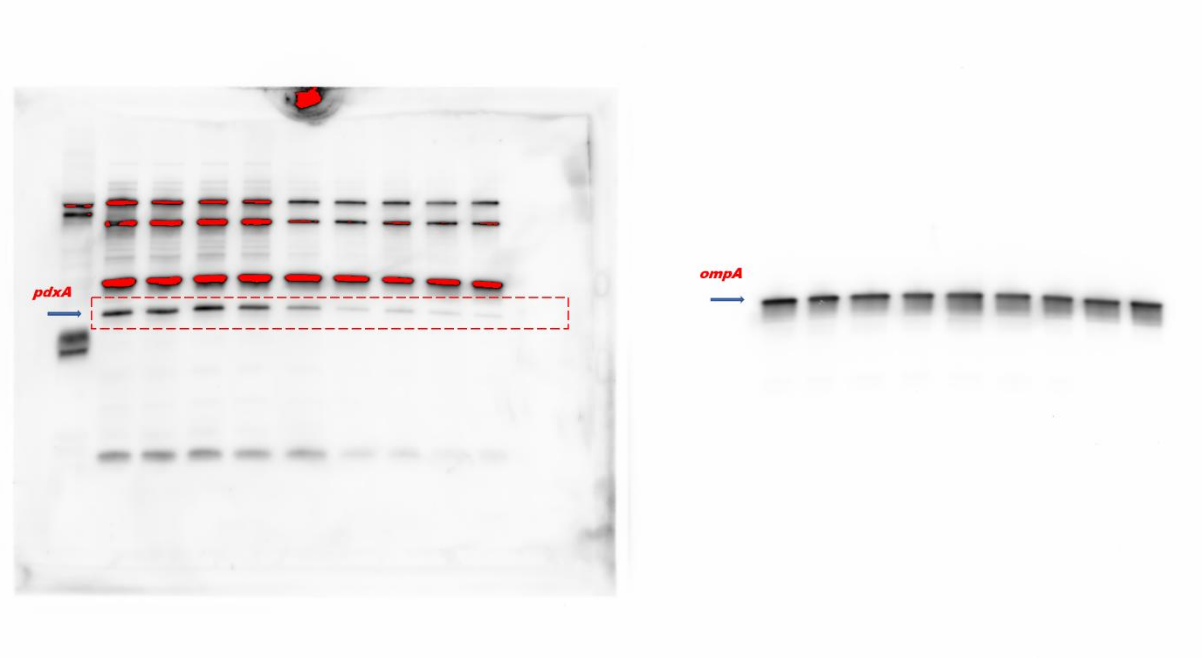

Supplement: Supplementary file 1 [file ijms-27-00584-s001.zip › ijms-4045466-supplementary.pdf]
